# Piezoresistive Soft Condensed Matter Sensor for Body-Mounted Vital Function Applications

**DOI:** 10.3390/s16030326

**Published:** 2016-03-04

**Authors:** Mark Melnykowycz, Michael Tschudin, Frank Clemens

**Affiliations:** 1 Laboratory for High Performance Ceramics, Empa, Swiss Federal Laboratories for Materials Science and Technology, Überlandstrasse 129, Dübendorf 8600, Switzerland; frank.clemens@empa.ch; 2 STBL Medical Research AG, Höh-Rohnenweg 6, Wilen 8832, Switzerland; michael.tschudin@stbl.ch

**Keywords:** piezoresistive, sensor, composite, hybrid, wearable computing, carbon black, pulse wave, polymer, motion capture

## Abstract

A soft condensed matter sensor (SCMS) designed to measure strains on the human body is presented. The hybrid material based on carbon black (CB) and a thermoplastic elastomer (TPE) was bonded to a textile elastic band and used as a sensor on the human wrist to measure hand motion by detecting the movement of tendons in the wrist. Additionally it was able to track the blood pulse wave of a person, allowing for the determination of pulse wave peaks corresponding to the systole and diastole blood pressures in order to calculate the heart rate. Sensor characterization was done using mechanical cycle testing, and the band sensor achieved a gauge factor of 4–6.3 while displaying low signal relaxation when held at a strain levels. Near-linear signal performance was displayed when loading to successively higher strain levels up to 50% strain.

## 1. Introduction

The current landscape of wearable computing products largely relies on the use of mass-produced sensors such as accelerometers, gyroscopes, temperature, *etc.* that can be easily integrated into designs such as fitness trackers and smart watches. Such sensors are all hard and inflexible, and therefore not easy to adjust to the surface of the human body. The advances in polymer-based sensor technology offer new solutions for flexible sensors mounted on the body for more advanced wearable sensor uses cases. Soft condensed matter sensor (SCMS) systems are a promising class of materials to provide flexible sensors for body-mounted wearable computing applications. The SCMS class of materials is proposed in order to define sensors which are derived from soft materials covered in condensed matter physics, but are soft given their higher mechanical flexibility, low material hardness (characteristic of polymers) and potentially non-linear mechanical behavior, which differs strongly from the behavior of sensors based on hard materials such as bulk silicon and metals.

Piezoresistive polymer monofilament sensor composed of carbon black (CB) mixed with an elastic matrix (e.g., elastomers or thermoplastic elastomer) is a good example of such soft condensed matter sensor materials because they can be elongated above 100% of their original length before rupture. Several articles have been published where such sensors are validated or compared [1,2]. The current work is focused on the implementation of the CB/TPE based SCMS into the design of a band sensor to measure mechanical strain of the surface of the human body, specifically when mounted on the wrist of a person. Depending on the mounting position on the wrist, it is possible to discern different movements of the hand/wrist, but also to detect the pulse-wave profile of blood moving through the radial artery, and from this to calculate the heart rate (HR) frequency of a person.

### 1.1. Conventional Sensor Design

Historically, the evolution of sensors has been heavily influenced by the integrated circuit (IC) design and manufacturing methods that have relied on surface and bulk micro machining of single crystal silicon wafers. This lead to the development of micro electrical mechanical systems (MEMS) and a variety of mass producible sensors types including accelerometers, atmospheric pressure, temperature, gyroscopes, *etc.* High volume and low cost of manufacturing has enabled the integration of sensors into low-cost electronics such as unmanned air vehicles (UAV), smartphones, smart watches, activity trackers, as well as open-hardware platforms such as the Arduino architecture design. The integration of accelerometers into wearable computing and smartphone products has allowed for direct use of device motion and orientation tracking which can be used at the application programming level to design a multitude of applications such as fitness and sleep tracking. The integration of light-based sensors and emitters such as light emitting diode (LED) and infrared (IR) can be used for pulse wave detection and heart rate (HR) frequency or blood oxygen detection by measuring the change in light absorption [3]. Basic motion detection and capture for device control is also possible by accessing accelerometer or gyroscope data for hand-mounted devices such as smart watches or motion capture bands such as the Myo^®^, which uses sensor fusion to connect the output of multiple skin-contact electrodes, accelerometers and gyroscopes to characterize hand and finger motions. All of these sensor types are based on IC production processes, and are therefore designed and built primarily with a layered design approach. The flat profile of sensor geometry means that they can be placed on the body, but cannot conform to or change their geometric share with the changing conditions of the human body, which are inherent given the viscoelastic nature of human skin [4,5,6].

### 1.2. Limitations of Conventional Sensors Design

The mounting of rigid sensors onto the body is extremely important because it defines the transfer of physical changes into sensor signals. Accelerometers for example should be flat against the surface or body to be able to monitor the XYZ components of movement correctly. IR or LED sensors placed on the skin with a deviation from a 90 degree mounting angle will significantly influence the light transmission and reception of the sensor, and negatively impact the ability to monitor the PW correctly. In pulse-wave oxymetry, motion artifacts (MA) pose a large problem in providing accurate heart rate results from the measured pulse-wave data and MA reduction techniques can be employed to improve sensor performance [7]. Additionally, it has been shown that the contact force with the skin of devices which measure photoplethysmographic (PPG) derived data can influence the pulse wave transit time (PTT) [8,9]. With regards to motion capture and monitoring, various systems including accelerometers placed at different positions on the body are used to characterize the relative change between accelerations in order to calculate the angular velocity of body parts relative to one another and compute significant biomechanical data, such as gait movement, arm positions, *etc*. The development of soft sensors which can conform to the surface of the body and the changing boundary conditions between the sensor and the contact surface with the human skin structure can allow new designs and concepts for new wearable computing applications and improve current problems with MA detection and removal in vital function monitoring applications.

### 1.3. Hand- and Textile-Mounted Sensors

Currently there are different options for hand or finger-mounted sensors that can capture finger movements. The commercially available piezoresistive FLEXPOINT Bend Sensor^®^ (www.flexpoint.com) is employed in the Mi.Mu Gloves [10] project, which seeks to allow control of electrical devices through hand and finger movements. In this design concept, bend sensors are placed over individual fingers, and as the fingers open or close, the resistance of the bend sensor changes, allowing for mapping of the Bend Sensor^®^ resistance to finger configuration [11]. In this case, the bend sensor only provides an approximate value for the finger orientation since one sensor covers the entire length of the finger. It is easy to cover the metacarpophalangeal (MCP) joint with the sensor placed at the back of the metacarpals and proximal phalanges. The Bend Sensor^®^ functions via the opening of cracks in the conductive ink of the sensor [12] which leads to an increase in resistance and is therefore related to the curvature of the bending deformation. However, to cover all the joints is not directly possible since the substrate of the Bend Sensor^®^ does not have the same flexibility as the human finger. This then leads to a general curvature of the sensor along the finger, but not for each individual joint.

For improved accuracy, it would be preferable to have a sensor monitor each joint and track the relative angle between finger sections. Additionally, problems may arise when integrating the bend sensor over a finger, which can undergo larger deformations than the bend sensor. Work by Matsuzaki and Tabayashi [13] have reported on metal sensors integrated in a silicon matrix which attain strains of 50% and can be integrated on a glove substrate to track individual sections of the fingers to provide better resolution of finger motion capture. Culha *et al.* [14] have demonstrated the use of CB/TPE monofilament sensors mounted on the fingers of gloves are able to discern between specific finger positions, with an applications proposed for interpreting American hand sign language. Mattmann *et al.* investigated motion capture on the body with the CB/TPE piezoresistive monofilaments, which were integrated with a form-fitted shirt and used to identify variations in body position of a person [15]. They demonstrated that the CB/TPE sensors can be strained up to 80% reversibly with a low hysteresis and no significant change in resistivity when changing motion speed. Additionally no significant change in sensor properties could be observed after several washing cycles. Capacitive strain sensors based on carbon nanotubes (CNT) electrodes layered on either side of a silicone elastomer layer have been reported by Cai *et al.* where strain gauges were able to stretch to 300%. Good linearity was achieved as well as repeatability over 1000 s of mechanical cycles at strains of 100%–200% [16]. Additionally, finger movements were discernable when mounted on a glove and different finger positions were performed. Hand position monitoring has also been investigated using commercially available FSR400 force sensitive resistors (FSR), which are based on electrode contact to a substrate coated with piezoresistive polymer [17]. With their WristFlex system, Dementyev and Paradiso [18] demonstrated that an array of 15 FSR 400 sensors mounted around the wrist can be used to detect finger pinch gestures in real-time.

Contactless sensors based on eddy or magnetic current induction monitoring is also an evolving area of sensor design for body motion and vital sign monitoring. An eddy current induction sensor includes a flat coil, which can be driven by an alternating current to send out an alternating magnetic field, which induces eddy currents in a conductive medium in close proximity to the device, which in turn creates an alternating magnetic field. The resulting interaction can be characterized and used as a contactless sensing system [19]. The primary physical feature of the eddy current sensor is a flat metallic coil, which can be easily integrated with textiles. Contactless sensors avoid the challenges posed by maintaining contact between the sensor surface and the human body, and additionally can be directly integrated into clothing. Teichmann *et al.* have reported on the MonitoRing system, which measured bioimpedance on the thumb, for an impedance plethysmography application. The system was able to monitor pulse at the fingertip, but other positions including the arm and the thumb base were not possible, with measurement errors attributed to motion artifacts resulting from muscle movements [20]. Later work by Teichmann *et al.* focused on the combined monitoring of respiration (using eddy current monitoring) and pulse (using photoplethysmography) using the FlexPock device, placed near the left breast pocket of a shirt [19]. In the MAIN shirt design, a magnetic induction sensor array (composed of four sensors) was used to detect both respiration and pulse using only the eddy current induction method [21]. Additionally, the recognition and classification of basic body motions (standing, sitting, slow/fast walking and jogging) have been demonstrated with magnetic induction monitoring an array of four sensors integrated with a textile shirt [22].

### 1.4. Polymer Piezoresistive Sensors

To a large extent, historically polymer strain sensor research has focused on conductive coatings on polymer monofilaments or woven substrates. This has resulted in the development of yarns with conductive fibers [23,24] or nanotube yarns [25], carbon or silver-coated polymer fibers [26]. Composite or hybrid materials have been developed via the integration of carbon nanotubes into a polymer matrix [27] or electroactive polymers with carbon fillers [28,29]. More recently, a pressure sensitive textile has been developed by coating Kelvar fibers with a SBS/AgNP composite, which allows for capacitive based pressure sensing in a woven fabric design [30]. The conventional conductive yarn design encompasses a sensor fiber element (for example a conductive wire) woven or twisted together with load-bearing and electrically insulating fibers. Such an approach appears to have been taken in implementation of Google Project Jacquard [31]. The conductive fiber could be metallic or carbon-coated [23,32], allowing sensor function integration with the structural fibers. The final yarn is a sensor that can support mechanical loads. Lee *et al.* developed a body-mountable stretchable strain sensor for motion detection by pattering silver nanoparticles on a polydimethylsiloxane (PDMS) substrate. Micro-cracks in the nanoparticle layer resulted in a resistance change at increasing strains, with a maximum strain of 20% achieved as well as mounting of the sensor separately on the fingers and on the wrist to discern basic movements [33]. Park *et al.* have utilized silver nano-particles in combination with elastomeric fibers to produce strain sensors that also function due to crack formation in the conductive nano-particle layer of the fibers. Strains of 140% were achieved, but with a high influence of fiber mat thickness on the resulting change in conductivity of the strain sensor.

Various approaches have also been employed with graphene for the creation of highly stretchable sensors. Yan *et al.* have reported on a stretchable graphene nanopaper which could achieve a strain of 100% with a linear strain region between 40%–70% strain [34]. Graphene-natural rubber composites were investigated by Boland *et al.* where graphene infused rubber bands were able to achieve a strain of 75% with repeatable resistance changes. Finger, arm, and throat mounted applications were presented in order to monitoring breathing, finger/arm motion, as well as blood pulse wave peaks [35].

### 1.5. Current Work: Band Sensor Design

The current works reports on the design and evaluation of a flexible band sensor structure, which includes the integration of CB/TPE piezoresistive sensor material with a stretchable textile band substrate. The band sensor was developed with the goal measuring the change in circumference of the wrist during basic hand motions. The band sensor utilized a piezoresistive polymer composite sensor [2] composed of conductive CB particles mixed together with an elastomer matrix (TPE). The advantage of using a TPE as the matrix material was the freedom of being able to thermal-form the sensor into various shapes by conventional polymer processing methods. A previous investigation focused on the monofilament sensor form [2], while an extruded ribbon geometry was used in the current work. The ribbon form could be used directly or further processed for specific sensor designs, in this case a U shape was chosen for the sensor, which was bonded to an elastic band substrate. This design was originally created in order to monitor expansion of the wrist for the calibration of a blood pressure monitor under development by STBL Medical Research AG. The current work focused on characterizing the strain sensor characteristics, and additionally, applications of the band sensor were investigated including motion capture of the hand and detection of the pulse-wave profile of the blood (with the band sensor mounted on the wrist). The investigation involved both laboratory mechanical testing as well as tests on human subjects.

## 2. Experimental Section

### 2.1. Band Sensor Production

The SCMS material was composed of Styrene-ethylene/buthylene-styrene (SEBS) triblock copolymer, produced by KRAIBURG TPE GmbH & Co. KG (Waldkraiburg, Germany), which was used as the matrix and carbon black produced by TIMCAL Graphite & Carbon, (Bodio, Switzerland) was employed as the filler. The carbon black particles had a density of 1.86 g/cm^3^ (measured by Helium Pycnometer) and a specific surface area of 61.48 m^2^/g (BET Nitrogen Surface Area measurement). The materials were mixed together using a torque rheometer (HAAKE Polylab OS, equipped with a HAAKE Rheomix OS high mixer chamber, Thermo Electron Corporation (Waltham, MA, USA) and type 600 Roller rotors. The mixing chamber was heated up to 180 °C and roller rotation was set to 10 rpm. The polymer was added in the chamber and mixed for five minutes, and then the carbon black was added. The components were then mixed until the torque reached steady-state conditions (approximately 1 h). The sensor was composed of 50 wt% CB and 50 wt% TPE. A previous investigation has shown that a mixture of 50 wt% CB and 50 wt% TPE represents an ideal balance between electrical and mechanical performance of the sensor material [36]. An increase of CB weight fraction will result in a decrease of the strain to rupture and an increase in the mechanical stiffness as well as an increase in electrical conductivity of the sensor.

**Figure 1:**
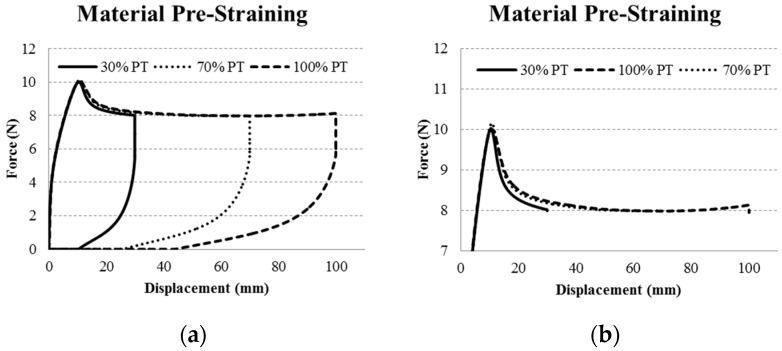
Pre-tension ribbon material evaluation, showing that the material has a minimum force response around 60% (**a**), and begins to increase starting at 80% (**b**), indicative of strain hardening.

The U-sensors were created by stamping a U shape out from bulk sensor material which had been extruded from feedstock material into a ribbon form and then pre-strained. The feedstock material was extruded into ribbon form using a Capillary Rheometer (Rosand RH7Flowmaster). A Niggeloh 200 bar pressure transducer was used to monitor the pressure of the melt through an orifice, and a rectangular opening (0.3 mm × 6.0 mm) at the end produced a ribbon during extrusion. The heating zones of the chamber were set to 190 °C and the feedstock material was placed into the bore. A shear rate of 1500/s was used to extrude the sensor ribbon, which was then pre-strained. In the pre-straining process, the mechanical yield point (20% strain) of the sensor material was exceeded so that the sensor response would be linear with an applied linear strain [2]. At 20% strain the thermoplastic component of the TPE will have yielded, and beyond that, the elastomer component plays a greater part in the tensile mechanical behavior of the material. A small study was performed on extruded ribbon structures to determine the ideal pre-strain range. Sensor ribbons were mounted in a Zwick Z005 tensile test machine (Zwick Roell Xforce P 200 N load cell, testExpert v11.1 software), with a gauge length of 100 mm between the clamps. Strains of 30%, 70%, and 100% were applied to separate samples (five samples per strain), and each strain was maintained for 60 s. The resulting force response is shown in Figure 1, where a plateau is seen between 50% and 70% strain, which is consistent with previous results [2]. In this strain region it is believed that the polymer chains of the material have been stretched but have not reached the strain hardening region. The 50%–70% strain range was then used for U-sensor production, as it would allow the material to stretch with good elasticity and provide a linear electrical response.

**Figure 2:**
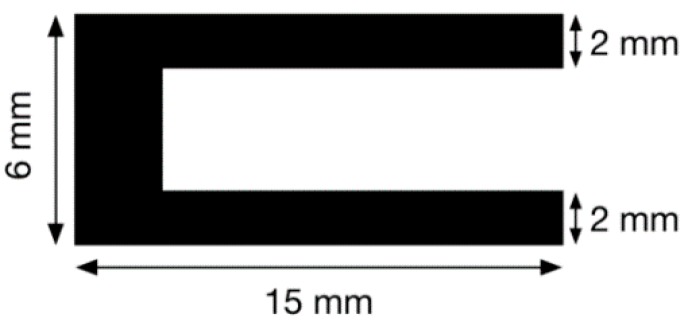
Dimensions of the U-shaped CB/TPE conducitve polymer sensor.

A U-shaped cutting die (LSB Stanzformen AG Gunzgen, CH) was used to cut out the U form from the extruded sensor ribbon. The final sensor geometry had an arm width of ~2 mm, and a length of 15 mm (as shown in Figure 2) The U-sensor (with bulk material pre-strain of 50%) was then bonded to an elastic band with liquid rubber treatments, forming the band sensor design.

**Figure 3:**
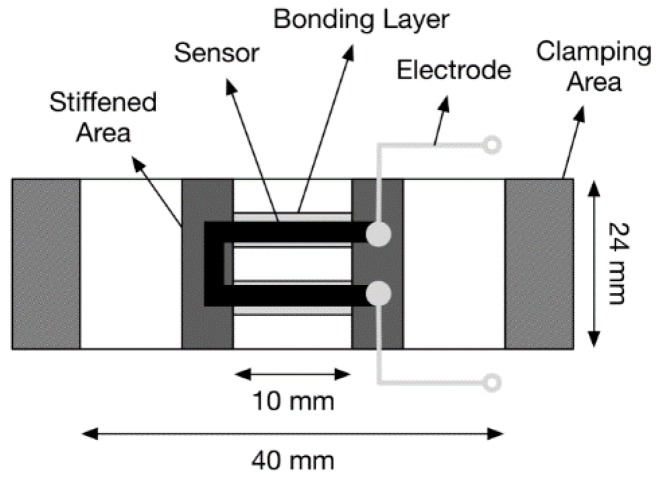
Schematic description of the textile band sensor tensile specimen; electrodes were connected at the ends of the U.

**Figure 4:**
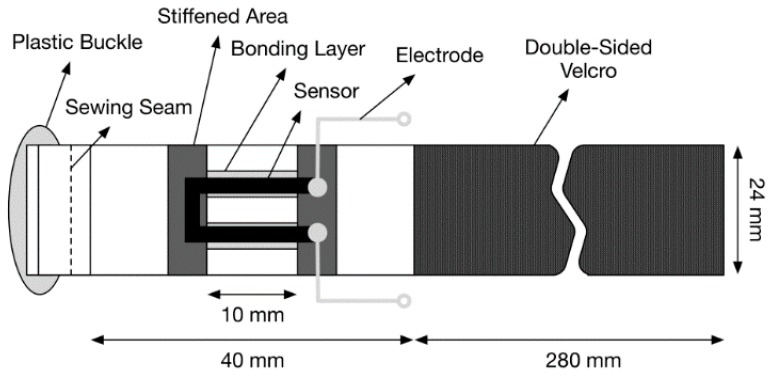
Schematic description of the wrist-mounted band sensor for motion and pulse wave capture.

Surface bonding was employed instead of woven integration since the integration of the U shape would be difficult with a monofilament or fiber in the textile band. A U shape was chosen in order to allow easy connection of the sensor to a smart watch module. The U shape allows the sensor to only connect on one side of a smart watch band, whereas a fiber placed along the band would require an extended conductive pathway back to the smart watch from the end of the sensor farthest from the smart watch. The bonding of the U-shaped sensor was done by STBL Medical Research AG with an adhesive coating used to apply the sensor to the elastic-band substrate. In order to reduce strain shielding and stress concentrations at the sensor ends, the STBL adhesive, as a thick liquid, was added to stiffen those regions and surround the area of quick drying glue. The thick liquid could be easily configured to different geometries by applying a mask during application. For the current study, sensors were produced for mechanical cycle testing (Figure 3), and for wearing on the wrist (Figure 4) for motion capture and pulse wave detection. The band sensor mounted on the wrist included Velcro and a plastic buckle for secure mounting, but the active area was the same as the tensile test specimen geometry.

### 2.2. Mechanical Tensile Testing

**Figure 5:**
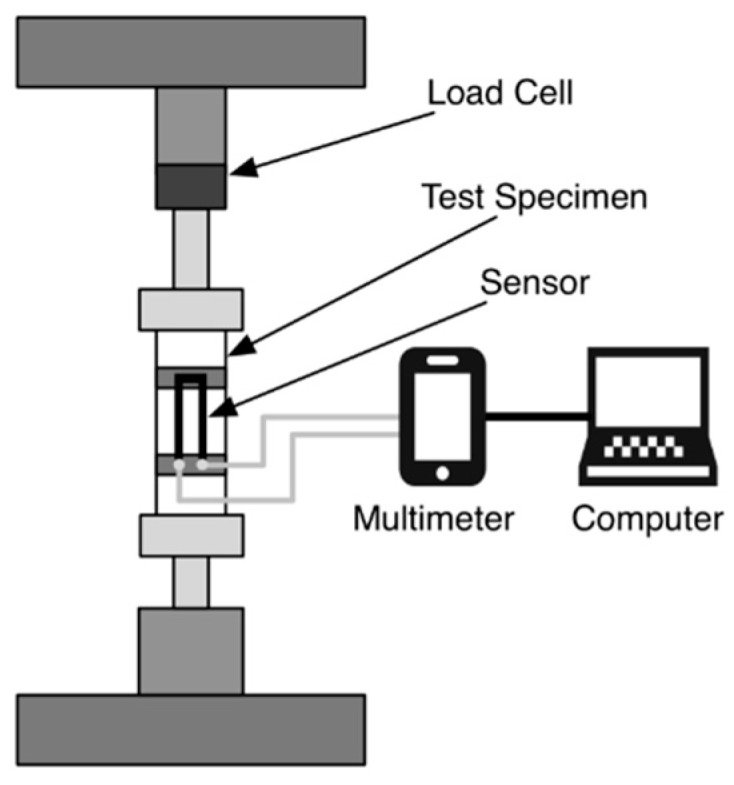
Tensile test setup for mechanical cycle testing.

Cyclic tensile loading was used to investigate the electrical-mechanical property coupling of the U-shaped textile sensor structure. A tensile testing machine (Zwick/Z005, Zwick Roell Xforce P 200N load cell, testExpert v11.1 software) was used to apply mechanical load, while a Metex M-3610D multimeter was used to record the resistance of the U-sensor in response to applied mechanical strain. ScopeView software was used with the multimeter to log the resistance measurements on a laptop computer. The test setup is depicted in Figure 5. The textile test specimen dimensions are shown in Figure 3, where the tensile gauge length was 40 mm, and the active length of the U-sensor was 10 mm.

The mechanical cycle test was displacement controlled and consisted of cycling with a 5% range between two different strain levels for 5 cycles with a hold of 1 min as each strain level. Each sample was subjected from 5% to 50% strain as shown in Table 1. The sample would be first brought to the maximum strain of the test (for example 50%), held for 1 min, and then brought to the strain minimum (45% in this example). After five cycles between the maximum and minimum strains (with 60 s held at each level), the sample was brought back to the starting point of the test (at 0% strain) before the next test commenced. A strain step of 50%–45% and 30%–25% with a 300 s holding time were also used to investigate signal stability over time. Three band sensors were evaluated in the mechanical cycle test. In total, 3 band sensors were produced and tested for the tensile test evaluation.

### 2.3. Band Sensor Mounting Positioning on the Wrist

**Figure 6:**
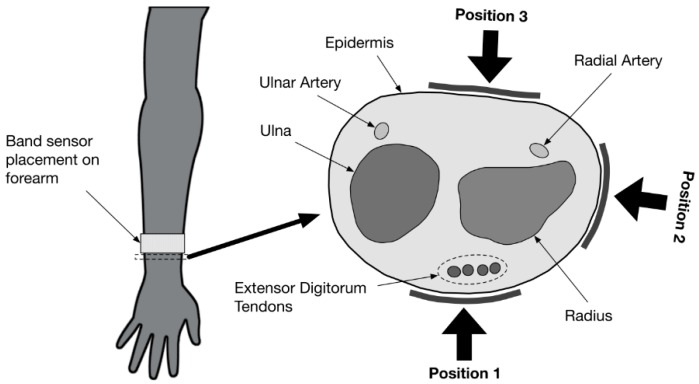
Cross-section schematic of the wrist area (extensor retinaculum/distal forearm) showing the band sensor mounting position for posterior (**P1**), radial (**P2**), and anterior (**P3**) mounting.

For mounting on the human wrist, band sensors with the design depicted in Figure 4 were produced. The wrist-mounted design included a buckle and double-sided Velcro^®^ so that the band sensor could be secured on the wrist, and deformation of the band was centralized on the sensor integration region. The optimal size of the buckle was printed in polylactic acid (PLA) by the fused filament fabrication (FFF) method using a Velleman K8200 3D printer. The pulse wave was measured with the band sensor tensioned around the wrist and placed at three positions: posterior, radial, and anterior (as shown in Figure 6). The sensor positions were chosen with reference to the standard anatomical position. The ulnar position was not used given the large surface change in the ulnar region on the wrist, which could lead to uneven loading of the U-sensor on different individuals. The anterior and posterior positions provided good coverage of the extensor and flexor tendons [37] respectively. The radial position would act well to detect rotation or twisting of the forearm as the radial bone would rotate relative to the ulna during such movement. In each case, the band sensor was mounted proximally to the extensor retinaculum tendon, such that the tendons of the extensor digitorum and extensor digiti minimi would be directly under the band sensor.

### 2.4. Pulse Wave Detection

The pulse wave of the blood was detected at the three positions around the wrist. The band sensor was connected to a smartwatch prototype developed by STBL Medical Research AG (Freienbach, Switzerland). Via the smart watch a current was applied to the band sensor and the resulting voltage across the sensor was recorded. A 24 bit analog-to-digital converter (ADC) was used in the design to provide a high resolution measurement of the band sensor signal with a data acquisition rate of 40 Hz. The smart watch prototype was connected via Universal Serial Bus (USB) connection to a laptop with a custom LabView^®^ program which ingested and saved the sensor data from the band sensor. To compare these measurements to a control value, a Polar FT60 with a H1 heart rate sensor chest belt was used to measure the heart rate during the test.

During the test, the subject sat in a chair with his hand on a table in front of him, the angle of his arm approximately 90° and in a relaxed state. The band sensor was secured around the wrist of the test subject so that it was tensioned but comfortable to wear. Data was acquired from the sensor for 5 min at each of the three sensor positions (P1, P2, and P3). Given the postion of the sensor with respect to the locaiton of the ulnar and readial arteries, it was expected that the posterior position (P3) would have the highest pulse wave signal.

### 2.5. Motion Detection Test Setup

During the motion detection analysis, 10 hand positions were defined and executed in succession, with a holding time at each position. The three sensor positions on the wrist (anterior, posterior, and radial) were used as with the pulse wave measurement. The sequence of hand positions is detailed with images in Table 2, while and the anatomical position targets for each hand position are detailed in Table 3. The STBL smart watch prototype was used to monitor and record the sensor signal with LabView^®^ software as previously described.

Each hand position was held for approximately 10 s before moving to the next position, so that the sensor was in a quasi-static state. This provided time for signal stabilization and was comfortable for the test subject. In developing the hand position test, the primary goal was to determine if specific tendon motions and bone orientations could be detected due to muscle excitations. This would form a basis for establishing development targets for improving body-mounted sensor function in future studies. Therefore, the hand position sequence began with the neutral position, and then included combinations of flexion, extension, supination and pronation of the hand, as well as adduction and abduction of the fingers.

The hand position test sequence was developed from the human anatomy perspective, but also with consideration to the human computer interaction (HCI) classification systems and research which have been developed for user input actions with regards to mobile computing and virtual user interface applications. Due to the evolution of mobile devices and touch-based input methods, user interaction patterns and gestures have been classified in order to have a library of finger motions and hand position which define discrete ways in which humans can interact with computing devices through touch. A library of unique touch interface gesture inputs (for flat interaction surfaces) has been established in order to create programming UI input libraries such as Gestureworks Core [38] to enable intuitive development of touch-based experiences on mobile devices or interactive walls by defining gesture inputs such as finger tapping, pinching, dragging, *etc.* The classification system has been extended to include devices with capabilities to track 3D motion and orientation. Gesture input classifications have been developed for specific wearable sensor devices including the Myo^®^ Armband, Nod Ring, and Nintendo Wiimote game controller [39]. These devices utilize an integrated IMU (Inertial Measuring Unit) in order to track relative changes in movement (x, y, z vector movement in 3D space) and rotation (x, y, z) of the device.

In conjunction with an internal ADC, the STBL prototype included a SCA3000 triple axis accelerometer (VTI Technologies Oy, Vantaa, Finland). Commercially available smart watch designs such as the Apple Watch [40] and Samsung Gear S2 include an IMU in their design. Therefore, tracking the response of the internal accelerometer and comparing it to the band sensor response of the STBL smart watch prototype would then allow for a basic indication of how a smart watch utilizing a standard IMU would compare to the performance of the band sensor. As has been shown with the WristFlex hand motion monitor [18], the ability to use sensor fusion and combine the senor output from an accelerometer with skin-mounted deformation sensors can result in expanded gesture monitoring.

Additionally, a finger movement test was done, where the hand was placed flat on a table, and each individual finger was raised slightly (extension) starting from the fifth digit (small finger) to the first digit (thumb) and back to the fifth digit. During the finger test, the palm remained in contact with the table, and the band sensor was in the posterior position. In the finger movement evaluation, only Position 3 (posterior) was used.

## 3. Results and Discussion

### 3.1. Band Sensor Signal Characterization

In the current work, the viscoelastic effects of the elastic band, elastic adhesive and elastic sensor material were not separated. Relaxation and drift of the bulk sensor material was previously evaluated in a monofilament fiber form [2] under successive loadings. Therefore, the current work focused on the sensor-band design in order to determine if signal relaxation (mechanical drift) existed in the system. In general, sensor drift is defined as a signal decrease over time (months or years), related to degradation of the physical properties of the sensor material. In elastomer based sensor materials, signal relaxation is used to describe the reduction in signal strength over a short time period (seconds or minutes). Signal relaxation is related to changes in the conductive network of CB particles related to the polymer chain orientation of the elastic matrix. Additionally, the elastic band substrate and the elastic adhesive will show a similar effect. All of these elastic components will certainly display a viscoelastic behavior in response to applied mechanical loads and the associated relaxation behavior (strain effect) will influence the measured resistance signal from piezoresistive sensor.

**Figure 7:**
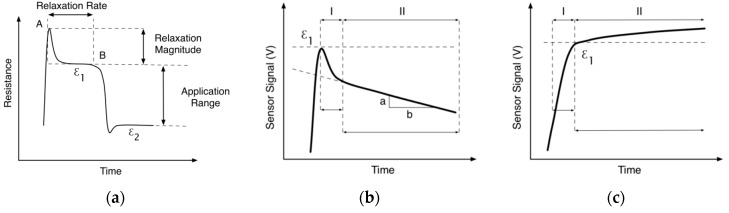
General behavior of the conductive polymer when a new strain level is applied (**a**); where an abrubt peak is seen, followed by a relaxation, a near-linear signal degradation is seen (**b**); In a slighlty under-loaded state (**c**) the signal gradually rises, attributed to the visoelastic nature of the integration materials.

During the tensile cycle test, the maximum signal occurred when the sensor attained the target strain level, followed by a relaxation or drift beyond that as shown in Figure 7a,b. This initial relaxation is assumed to be related to the intrinsic characteristics of the sensor material, but also to the mechanical relaxation behavior of the elastic band and adhesives used in the Stiffened Area of the band sensor design (Figure 3). From past research [2] it is known that there generally exists a nonlinear degradation shortly after the target strain is achieved, and then the signal degradation is near-linear and a much smaller than the initial non-linear degradation and can reach a constant value. Logically, as the Stiffened Area material relaxes, less strain is transferred to the sensor region and the signal would decrease. Alternatively, the sensor material might be slightly under-loaded (Figure 7c), where the sensor signal rises after attaining the target strain level, this is attributed to loading of the Bonding Layer material (Figure 4), which bonds the sensor to the elastic band substrate. As this material relaxes, more strain is transferred to the sensor region, resulting in a signal increase.

**Figure 8:**
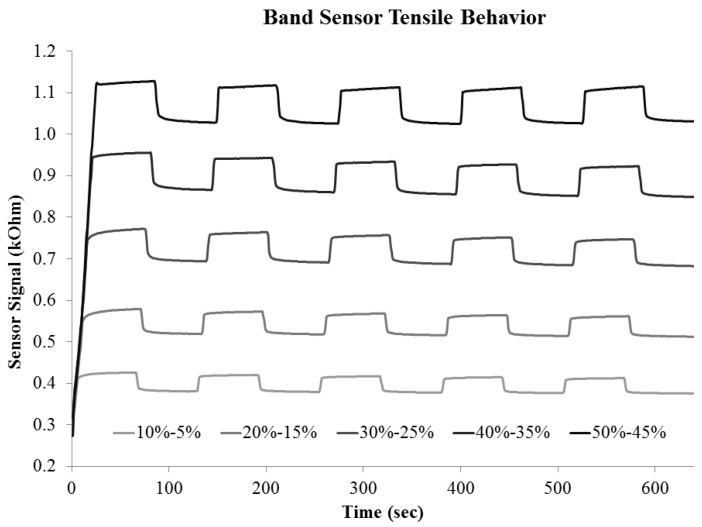
Characteristic band sensor response to tensile strain cycle test. Each sample was first brought to the maximum strain followed by 5% strain range cycling test.

This model for the loading of the band sensor and the signal behavior is represented in the experimental data of Figure 8, where a change in the signal character is seen at the higher strain level. Under-loading behavior is seen in the strain step data between 5% and 30%, while the higher strains of 40%–50% have a more stable signal at the target strain level. In both cases, this behavior is not seen in the subsequent strain cycles (2nd—5th), suggesting that the materials in the band sensor have reached a stable state after initial loading.

**Figure 9:**
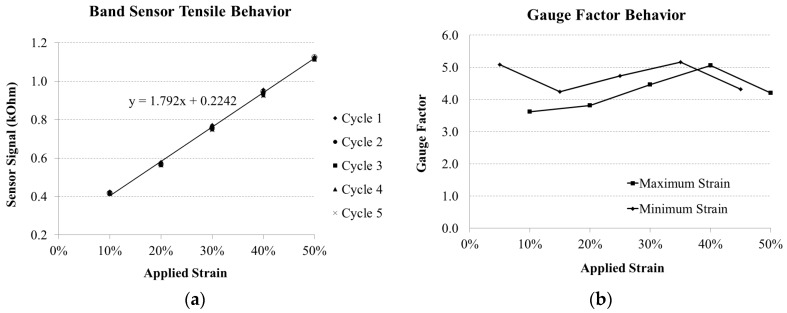
Characteristic band sensor signal tensile strain behavior (**a**) and gauge factor (**b**). The gauge factor is reported for the maximum (ε_1_) and minimum (ε_2_) strain levels as shown in Figure 7b.

The combined sensor signals taken at point B (Figure 7a) for each strain step (10%–50% strain) are plotted together in Figure 9a, where a linear character is seen along the loading path. This behavior is consistent with monolithic filament tests from the previous work [2], where linear behavior was seen in the tensile loading of 0.300 mm diameter monofilaments starting from a strain of 20%, and continuing until approximately 150%. Since the U sensors were pre-strained to 50% in the current work, they operate well within the linear signal response range of the bulk sensor material. Given the 50% pre-strain of the current band sensor, these results can be compared with the equivalent monofilament strain range of 50%–100%.

The gauge factor (GF) is defined in Equation (1), and can be calculated using data from the tensile test results. The GF is a characterization of the sensitivity of the band sensor as a strain sensor, and was calculated for each strain step, using the initial resistance, (R_O_) of each sensor before each test started strain was applied while R is defined as the resistance at point B as defined in Figure 7a. The three tested band sensors had R_O_ values of 0.298, 0.291, and 0.596 kOhm respectively.

(1) $$GF=\frac{\frac{R-R_{o}}{R_{o}}}{\frac{l-l_{o}}{l_{o}}}=\frac{\frac{\Delta R}{R_{O}}}{\varepsilon }$$

The GF results are displayed in Figure 9b, where the average values calculated from the total test specimens are reported. The GF results are relatively stable, with a small change over the applied strain range, but fall within 4–5 from 10% to 50% strain. The large GF value at 5% is likely related to the composite layering of the band sensor design, with different shielding effects occurring at different strain ranges with regards to the bonding layer between the sensor and the band as well as the stiffening regions (Figure 3) and the sensor ends. The range of GF values over different samples and tests was between 4 and 6.4, which is in the same order of magnitude of metal foil strain gauges (GF between 2 and 5) [41]. The results also correspond to strain gauge sensors based on a styrene-butadiene-styrene matrix with multi-walled carbon nanotubes (MWCNT), which have GF results of 4–10 at 20% strain [42]. The change in GF over the strain range is possibly attributed to the effects of yielding of the different viscoelastic layering materials over the strain range. This would result in a difference in relaxation behavior of the different stiffness regions of the band sensor, and therefore the relative strain between integration regions and the sensor region would not be proportionally equal to one another over the entire strain range.

**Figure 10:**
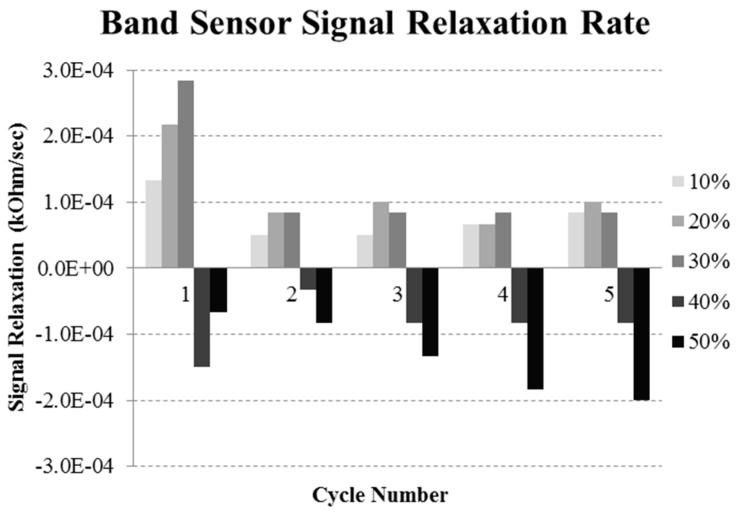
Band sensor relaxation rate as a function of test cycle number and applied strain range. The rate is defined as the signal relaxation between points A and B, divided by the time in seconds as described in Figure 7a.

**Figure 11:**
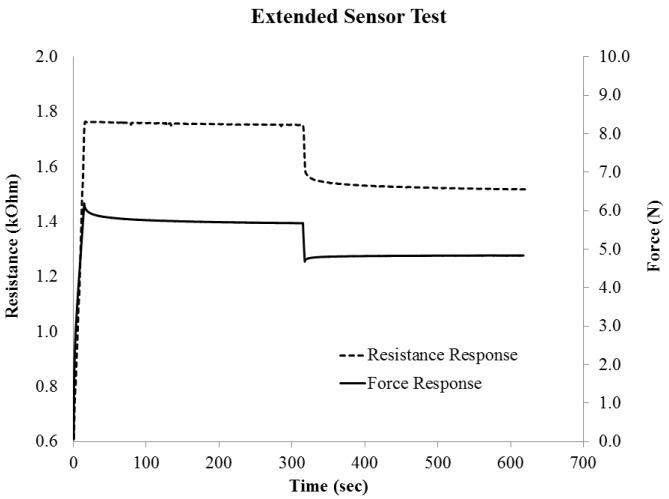
Characteristic band sensor tensile strain behavior held at 30% and then 25% strain for 5 min at each strain level.

The signal relaxation rate results are plotted in Figure 10, in order to show how the signal changed when held at each strain level. The most severe signal change was in the first loading cycle. In general the absolute drift values were less than 0.0002 kOhm/s, over a holding time of 1 min. When taken as a percentage of the initial sensor value, the maximum drift loss was 0.1%–0.4%/s for the stretch bands. The gauge factor was high on the first cycle of each test, and was also high for smaller rather than largest strains. The tensile test investigation showed the stretch bands to have low drift, and when stabilized at a strain level, the drift was near to zero. The characteristic extended strain holding test results are shown in Figure 11, with both the sensor signal and the force response are displayed. Here the band sensor was strained to 30% and then 25%, and held for 300 s at each level. The force response shows the relaxation response to the 30% strain, while the sensor signal is nearly flat. At the 30% strain step, the relaxation was 0.0069%/s over the 300 s of the strain step.

### 3.2. Band Sensor Motion Capture

**Figure 12:**
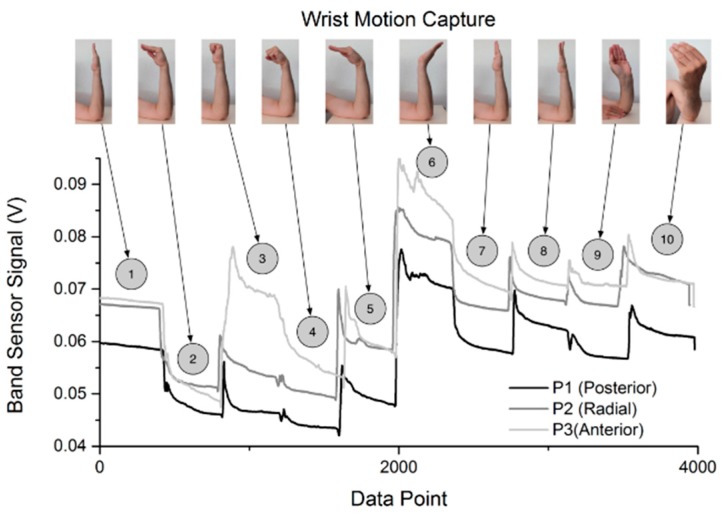
Motion capture of the three band sensor wrist positions (posterior, radial, anterior) displayed together.

The band sensor motion capture evaluation was done by utilizing the three defined positions (posterior, radial, anterior) around the wrist and performing 10 specific hand movements (shown in Figure 12). Additionally, a test was completed with the STBL smart watch prototype mounted on the wrist and the band sensor at the anterior position. The characteristic motion capture sensor behavior is shown in Figure 12, where the three position results from three separate tests are plotted together. The band sensor was able to discern the different movements with good responsiveness and sensitivity. When highly tensioned (such as with movement 6) the signal would first peak and then undergo a relaxation, similar to, but more severe than the behavior of tensile measurements. Small changes in the signal are attributed to the unstable nature of performing the hand exercises, since small movements would be registered by the band sensor. The relaxation in the signal is taken to be partially a function of the elastic band and adhesive materials used in the sensor design. However, the viscoelastic nature of the skin and tissue structure of the test subject (in this test, a 37 year old male) has to be taken into account too. Therefore the relaxation during the test analyses was more complex than for the pure piezoresistive sensor. The magnitude of the relaxation was much greater than that seen in the controlled tensile cycle tests with the sensor band structure. This is in good agreement with the expected viscoelastic behavior of the skin, which is a collection of collagen and elastin and is considered to be a viscoelastic material [43]. Furthermore, it has been shown that the arm displays a viscoelastic relaxation when a vertical force is applied [6]. During the test, movements 1 and 7 were both in the neutral position and should have displayed a similar response. Although the levels of P1 and P2 were comparable, P3 was higher than expected in the movement 7. The difference may have been due to slippage of the band sensor over the wrist, since movement 6 placed the largest strain on the band sensor and may have resulted in a modification of the interface pressure between the band sensor and wrist surface. In the transition between movements 3 and 4, there is a small difference in P1 and P2, but a large change in P3. This is consistent with the structure of the arm, since the transition from movement 3 to 4 requires that flexion occur in the hand, and the tendons on the anterior side of the wrist must relax.

In a future work, it would be feasible to develop an algorithm to use the relative sensor outputs together in order to define specific hand positions (or gestures), given the relative change in resistance values at each position for each hand movement. Additionally, a new design for the band substrate which conforms better to the skin surface may improve the problems attributed to band slippage. Finally, reducing the sensor size and adding multiple sensors to a single band would allow for a more robust band sensor design.

**Figure 13:**
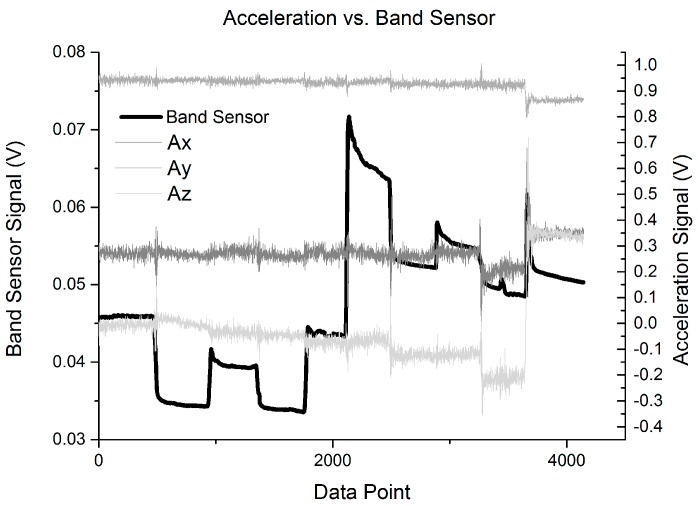
Combined results of the band sensor in the posterior position alonside the STBL smartwatch prototype with 3-axis accelerometer.

To compare the band sensor with current wearable sensor designs (fitness tracker bands and smart watches) a hand motion test was done with the STBL smart watch prototype on the wrist alongside the band sensor. The accelerometer data was recorded to compare with the band sensor. In this evaluation, the band sensor was placed just above the smart watch prototype on the wrist, so that the accelerometer would be close to the band sensor position. During the 10 hand position test, small changes could be seen in the accelerometer data (Figure 13). However, these were quite small and generally difficult to separate from the noise floor of the sensor. Conversely, the band sensor showed a much larger response, and was strongly responsive to the different hand positions.

Figure 13 shows that the band sensor can provide a new dimension of motion capture as compared with vital function motion artifact reduction approaches, which reply solely on accelerometer sensor data to correct motion artifacts in the heart rate data acquired with photo plethysmograph (PPG) signals [7]. For specific motion artifact reduction or gesture applications, the accelerometer and band sensor signal data sets can be combined in order to benefit from the strengths of both sensor types, as was shown to be effective with the WristFlex system to monitor hand gestures [18].

**Figure 14:**
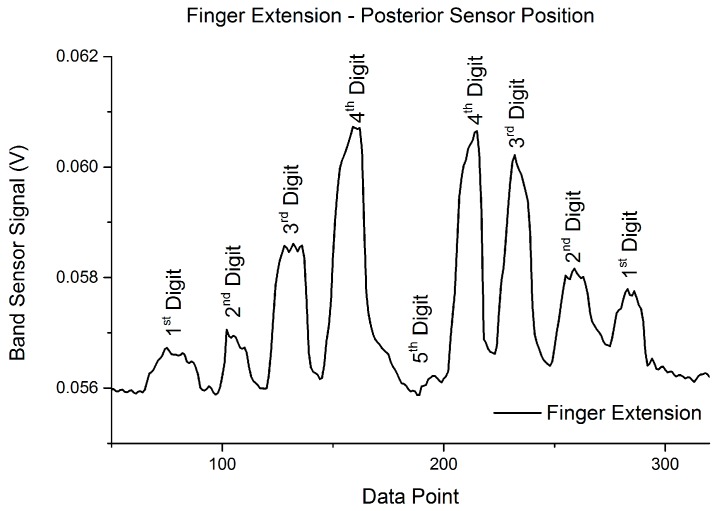
Finger extension motion capture test, sensor in the P1 (posterior) position on the wrist, covering the tendons of the extensor digitorum and digiti minimi.

The characteristic digit extension test results are shown in Figure 14, where it is seen that each digit can be discerned by individual signal peaks with the exception of the first digit (thumb). This result is logical given the sensor mounting position (posterior) and the anatomy of the hand. Extension of the fifth digit is governed by the tendons of the extensor pollicus longus and pollicus brevis, which are both on the radius side of the wrist, and therefore not captured when the band sensor is in the posterior position. The extension of the digits is governed by the tendons of the extensor digitorum and digiti minimi, which are all directly under the band sensor while mounted in the posterior position. This controlled test showed that the individual finger movement could be discerned based on the relative change in sensor signal between movements.

### 3.3. Pulse Wave Detection

The band sensor was originally designed to monitor the expansion of the wrist, and pulse wave detection was seen as an added benefit for this particular sensor configuration. From a sensing viewpoint, the pulse wave is essentially a perpendicular force leading to a slight increase in the tension of the band. The tension of the band will result in a tension in the sensor which will lead to an increase in electrical resistance, and therefore monitoring of the pulse is seen as being related to deformation of the band. During bending the increase in strain on one side of the sensor leads to the electrical resistance increase, which would be consistent with classical beam theory. The positioning of the band sensor around the wrist showed a strong dependence on optimal sensor signal quality. Additionally, modification of contact between the sensor and the surface of the skin disrupted the pulse wave signal capture.

**Figure 15:**
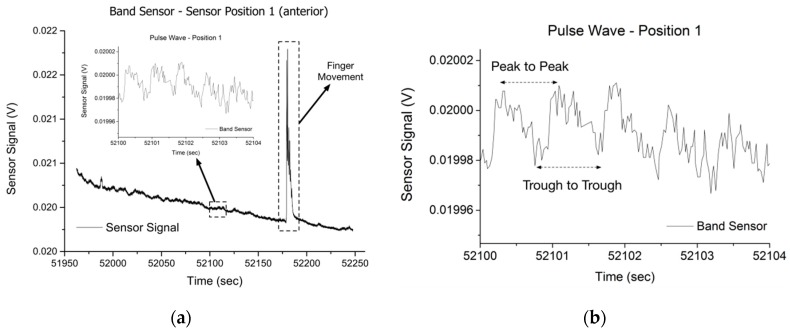
Raw data from the Position 1 test is displayed (**a**), detailing the difference between the stable signal and when the fingers are flexed. The stable area (**b**) was used for pulse wave analysis.

**Figure 16:**
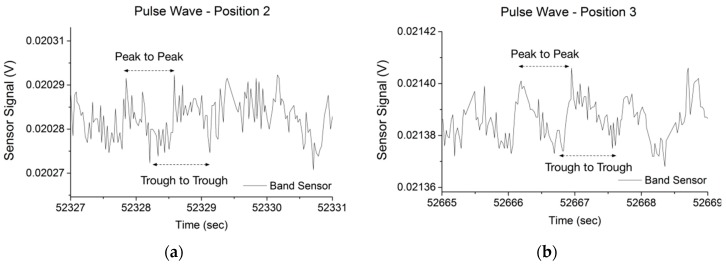
Band sensor pulse wave results for Position 2 (**a**) and Position 3 (**b**).

The raw band sensor data from the position 1 (posterior) trial is shown in Figure 15. The heart rate was determined by analyzing the wave profile in stable regions of the test. The test subject was directed to flex his fingers slightly in the latter part of the test, and the resulting signal disruption is highlighted. The characteristic pulse wave signals from Position 2 and 3 are detailed in Figure 16. Position 1 and 3 produced a pulse wave profile with easily discernable peaks, while at Position 2 it was not possible to clearly define peaks or troughs consistently, although adjacent peaks could be identified to calculate the heart rate. The poor performance at Position 2 was expected, since the sensor was positioned over the radial bone, and the mass of the radial was expected to shield the mechanical pulse wave from reaching the band sensor. When placed at Position 1 (posterior) or at Position 3 (anterior) side of the wrist, the pulse wave was discernible with both the peaks and troughs evident (Figure 16), which corresponded to the systolic and diastolic pressures respectively [44]. After identification of the peaks and troughs it was straightforward to calculate the beats-per-minute (BPM) and determine the heart rate. The BPM was calculated from the peak-to-peak and trough-to-trough of the raw data from eight points along the pulse wave data of Position 1 and 3. The test subject wore a Polar HR Monitor chest strap sensor for basic correlation of the calculated BPM to validate the results. At Position 1 the peak-to-peak calculation resulted in 79.9 BPM and the trough-to-trough was 77.3 BPM. At Position 3 the peak-to-peak value was 75.1 BPM and the trough-to-trough was 77.9 BPM, while the Polar HR Monitor (Polar FT60 training computer with H1 heart rate sensor) reported 76–80 BPM over the course of the test. Although pulse wave peaks corresponding to the systolic and diastolic blood pressure were easily discernable during the test, it was shown that finger movement (Figure 15) would easily disrupt the sensor signal. Since Position 1 and 3 were directly over the tendons structures of the wrist, flexion or extension of the hand fingers would lead to the tendons applying pressure on the skin structure and the contact between the skin surface and the band sensor would be modified.

## 4. Conclusions

The current work has shown the ability to build a band sensor for strain monitoring using an SCMS based on CB/TPE hybrid material, which can act as a body-mounted motion capture and blood pulse-wave monitoring sensor. When used as a motion capture sensor on the human wrist, the fusion of different sensor positions displayed the ability of identifying discrete hand positions and arm rotation. This was possible since the band sensor is sensitive to the movement of tendons in the wrist, which are not easily captured by conventional sensors such an accelerometer mounted in a smart watch form factor. It was shown that the wrist-mounted band sensor is additionally sensitive enough to track the blood pulse wave of a person, allowing for the determination of pulse wave peaks corresponding to the systole and diastole blood pressures in order to calculate the heart rate. Under mechanical cycle testing, the band sensor achieved a gauge factor of 4–6.3 and low signal relaxation when held at specific strain levels.

Although the band sensor was shown to be sensitive enough to act as a motion and pulse wave monitor on the wrist, further development can optimize the band sensor to the specific applications and improve sensor performance. A critical design direction will be the improvement of contact between the band sensor and the human body, so that slippage of the sensor during movement can be reduced or eliminated. Furthermore, by reducing the sensor size and adding multiple sensors to the band, it would be possible to target local areas of deformation on the wrist and potentially separate bone rotation from tendon movement. In the next phase of development the plan is to investigate the integration of layered manufacturing methods with the sensor and band design in order to optimize the band and to investigate different sensor applications on the human body.

## Figures and Tables

**Table 1:** Strain ranges applied to the test specimens.

Strain Test 1	10%–5.0%	20%–15%	30%–25%	40%–35%	50%–45%	35%–15%
Hold Time	60 s	60 s	60 s	60 s	60 s	60 s
Strain Test 2	50%–45%	30%–25%				
Hold Time	300 s	300 s				

**Table 2:** Images of hand positions defined for the motion detection evaluation.

1	2	3	4	5	6	7	8	9	10
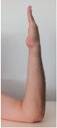	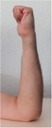	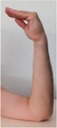	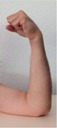	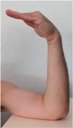	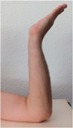	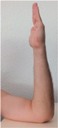	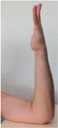	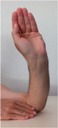	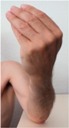

**Table 3:** Anatomical description of hand positions definitions.

Hand Position	Description
1	Neutral, finger adduction
2	Finger flexion
3	Palmar flexion (~30°) and finger flexion (~30°)
4	Palmar flexion (~30°) and full finger flexion
5	Palmar flexion (~30°) and finger flexion (~45°)
6	Hand extension
7	Neutral, finger adduction
8	Finger abduction
9	Hand pronation, finger adduction, 1st and 5th digit opposition
10	Hand supination, finger adduction, 1st and 5th digit opposition

## References

[1] De Rossi D., Della Santa A., Mazzoldi A. (1999). Dressware: Wearable hardware. Mater. Sci. Eng. C.

[2] Melnykowycz M., Koll B., Scharf D., Clemens F. (2014). Comparison of Piezoresistive Monofilament Polymer Sensors. Sensors.

[3] Fontaine A., Koshi A., Morabito D., Rodriguez N. (2013). Reflectance-Based Pulse Oximetry for the Chest and Wrist.

[4] Nemoto T., Isogai Z., Koide K., Itoh Y., Nogata F., Shimamoto A., Ju D.Y., Matsuura H. Evaluation of dermal segment on viscoelasticity measurement of skin by rheometer. Proceedings of the SPIE, Health Monitoring of Structural and Biological Systems.

[5] Vexler A., Polyansky I., Gorodetsky R. (1999). Evaluation of skin viscoelasticity and anisotropy by measurement of speed of shear wave propagation with viscoelasticity skin analyzer. J. Investig. Dermatol..

[6] Kwon H.J., Kwon Y.H., Kim Y.H. (2006). Biomechanical skin measurement system for anlaysis viscoelasticity. Key Eng. Mater..

[7] We P., Guo R., Zhang J., Zhang Y.T. A new wristband wearable sensor using adaptive reduction filter to reduce motion artifact. Proceedings of the International Conference on Information Technology and Applications in Biomedicine.

[8] Teng X.F., Zhang Y.T. (2004). The effect of contacting force on photoplethysmographic signals. Phys. Meas..

[9] Teng X.F., Zhang Y.T. (2006). The effect of applied sensor contact force on pulse transit time. Phys. Meas..

[10] Mi.Mu Gloves Project. http://dev-blog.mimugloves.com/.

[11] Simone L.K., Kamper D.G. (2005). Design considerations for a wearable monitor to measure finger posture. J. NeuroEng. Rehabil..

[12] Flexpoint Sensor Systems (1997). Bend Sensor Technology Mechanical Application Design Guide.

[13] Matsuzaki R., Tabayashi K. (2015). Highly Stretchable, Global, and Distributed Local Strain Sensing Line Using GaInSn Electrodes for Wearable Electronics. Adv. Funct. Mater..

[14] Culha U., Nurzaman S.G., Clemens F., Iida F. (2014). SVAS(3): Strain Vector Aided Sensorization of Soft Structures. Sensors.

[15] Mattmann C., Clemens F., Tröster G. (2008). Sensor for Measuring Strain in Textile. Sensors.

[16] Cai L., Song L., Luan P., Zhang Q., Zhang N., Gao Q., Zhao D., Zhang X., Tu M., Yang F. (2013). Super-stretchable, Transparent Carbon Nanotube-Based Capacitive Strain Sensors for Human Motion Detection. Sci. Rep..

[17] FSR 101—THE BASICS. http://www.sensitronics.com/fsr101.htm.

[18] Dementyev A., Paradiso J.A. WristFlex: Low-power gesture input with wrist-worn pressure sensors. Proceedings of the 27th Annual ACM Symposium on User Interface Software and Technology.

[19] Teichmann D., De Matteis D., Walter M., Leonhardt S. A Bendable and Wearable Cardiorespiratory Monitoring Device Fusing Two Noncontact Sensor Principles; In Proceedings of the 2014 11th International Conference on Wearable and Implantable Body Sensor Networks (BSN).

[20] Teichmann D., Foussier J., Löschcke D., Leonhardt S., Walter M. (2013). MonitoRing—Magnetic induction measurement at your fingertip. J. Phys. Conf. Ser..

[21] Teichmann D., Kuhn A., Leonhardt S., Walter M. (2014). The MAIN Shirt: A Textile-Integrated Magnetic Induction Sensor Array. Sensors.

[22] Teichmann D., Kuhn A., Leonhardt S., Walter M. (2013). Human motion classification based on a textile integrated and wearable sensor array. Clin. Phys. Physiol. Meas..

[23] Huang C.-T., Shen C.-L., Tang C.-F., Chang S.-H. (2008). A wearable yarn-based piezo-resistive sensor. Sens. Actuators A Phys..

[24] Huang C.-T., Tang C.-F., Lee M.-C., Chang S.-H. (2008). Parametric design of yarn-based piezoresistive sensors for smart textiles. Sens. Actuators A Phys..

[25] Zhao H., Zhang Y., Bradford P.D., Zhou Q., Jia Q., Yuan F.-G., Zhu Y. (2010). Carbon nanotube yarn strain sensors. Nanotechnology.

[26] Gibbs P., Asada H.H. (2005). Wearable Conductive Fiber Sensors for Multi-Axis Human Joint Angle Measurements. J. NeuroEng. Rehabil..

[27] Martinez F., Obieta G., Uribe I., Sikora T., Ochoteco E. (2009). Polymer-Based Flexible Strain Sensor. Procedia Chem..

[28] Cochrane C., Koncar V., Lewandowski M., Dufour C. (2007). Design and Development of a Flexible Strain Sensor for Textile Structures Based on a Conductive Polymer Composite. Sensors.

[29] Shui X., Chung D.D.L. (1996). A piezoresistive carbon filament polymer-matrix composite strain sensor. Smart Mater. Struct..

[30] Lee J., Kwon H., Seo J., Shin S., Koo J.H., Pang C., Son S., Kim J.H., Jang Y.H., Kim D.E. (2015). Sensors: Conductive Fiber-Based Ultrasensitive Textile Pressure Sensor for Wearable Electronics. Adv. Mater..

[31] Google Project Jacquard. https://www.google.com/atap/project-jacquard/.

[32] Flandin L., Chang A., Nazarenko S., Hiltner A., Baer E. (2000). Effect of strain on the properties of an ethylene-octene elastomer with conductive carbon fillers. J. Appl. Polym. Sci..

[33] Lee J., Kim S., Lee J., Yang D., Park B.C., Ryu S., Park I. (2014). A stretchable strain sensor based on a metal nanoparticle thin film for human motion detection. Nanoscale.

[34] Yan C., Wang J., Kang W., Cui M., Wang X., Foo C.Y., Chee K.J., Lee P.S. (2014). Highly Stretchable Piezoresistive Graphene-Nanocellulose Nanopaper for Strain Sensors. Adv. Mater..

[35] Boland C.S., Khan U., Backes C., O’Neill A., McCauley J., Duane S., Shanker R., Liu Y., Jurewicz I., Dalton A.B. (2014). Sensitive, High-Strain, High-Rate Bodily Motion Sensors Based on Graphene–Rubber Composites. ACS Nano.

[36] Clemens F.J., Koll B., Graule T., Watras T., Binkowski M., Mattmann C., Silveira I. (2013). Development of Piezoresistive Fiber Sensors, Based on Carbon Black Filled Thermoplastic Elastomer Compounds, for Textile Application. Adv. Sci. Technol..

[37] Ronan O’Rahilly F.M. (2008). Stanley Carpenter, Basic Human Anatomy.

[38] Gestureworks Core. http://gestureworks.com/.

[39] GestrueML—Sensor Gesture Index. http://www.gestureml.org/doku.php/gestures/sensor/gesture_index.

[40] Chipworks Apple A1554 Smart Watch Basic Product Teardown Report. http://www.chipworks.com/competitive-technical-intelligence/overview/technology-reports/recent-reports/download-the-apple.

[41] HBM Hottinger Baldwin Messtechnik GmbH (HBM). http://www.hbm.com/.

[42] Costa P., Silvia C., Viana J.C., Lanceros Mendez S. (2014). Extruded thermoplastic elastomers styrene-butadiene-styrene/carbon nanotubes composites for strain sensor applications. Compos. Part B Eng..

[43] Silver F.H., Freeman J.W., Devore D. (2001). Viscoelastic properties of human skin and processed dermis. Skin Res. Technol..

[44] Nelson M.R., Stepanek J., Cevette M., Covalciuc M., Hurst R.T., Tajik A.J. (2010). Noninvasive Measurement of Central Vascular Pressures With Arterial Tonometry: Clinical Revival of the Pulse Pressure Waveform?. Mayo Clin. Proc..

